# Traditional Chinese medicine (Xielikang) reduces diarrhea symptoms in acquired immune deficiency syndrome (AIDS) patients by regulating the intestinal microbiota

**DOI:** 10.3389/fmicb.2024.1346955

**Published:** 2024-02-16

**Authors:** Pengfei Meng, Guichun Zhang, Xiuxia Ma, Xue Ding, Xiyuan Song, Shuyuan Dang, Ruihan Yang, Liran Xu

**Affiliations:** ^1^Henan University of Chinese Medicine, Zhengzhou, China; ^2^The First Affiliated Hospital of Henan University of Chinese Medicine, Zhengzhou, China

**Keywords:** intestinal microbiota, immunity, HIV, AIDS, 16S rRNA gene

## Abstract

Diarrheal acquired immune deficiency syndrome (AIDS) seriously affects the quality of life of patients. In this study, we analyzed the differences in the intestinal microbiota among healthy individuals, AIDS patients without diarrhea and AIDS patients with diarrhea through high-throughput sequencing. The microbial diversity in the intestines of patients in the AIDS diarrhea group was significantly increased, and after treatment with Xielikang, the intestinal microbial diversity returned to the baseline level. At the phylum level, compared those in to the healthy (ZC) and AIDS non diarrhea (FN) groups, the relative abundances of Bacteroidetes and Verrucomirobia in the AIDS diarrhea (FA) group before treatment were significantly increased, while the relative abundance of Firmicutes was significantly decreased. Similarly, compared with those in the FA group, the relative abundances of Bacteroidea and Firmicutes in the AIDS diarrhea (FB) group after treatment were significantly increased, while the relative abundance of Firmicutes was significantly decreased after treatment. Additionally, there was no significant difference between the ZC and FN groups. At the genus level, compared with those in the ZC group, the relative abundance of *Prevotella* and *Escherichia_Shigella* in the FA group was significantly increased, while the relative abundances of *Megamonas* and *Bifidobacterium* was significantly decreased compared to that in the ZC group. After treatment with Xielikang, the relative abundance of *Prevotella* and *Escherichia_Shigella* in the FB group were significantly decreased, while the relative abundances of *Megamonas* and *Bifidobacteria* were significantly increased than those in the FA group; moreover, there was no significant difference between the ZC and FN groups. The functional prediction results showed that the ketodeoxyoctonate (Kdo) transfer to lipid IVA III and the superpathway of N-acetylglucosamine pathways in the AIDS diarrhea group were significantly altered. The correlation analysis results showed that *Dorea* was positively correlated with inflammatory factors, while *Streptococcus* and *Lactobacillus* were negatively correlated with inflammatory factors. The composition and function of the intestinal microbiota changed significantly in AIDS diarrhea patients, which affected the immune function of the host. The Xielikang capsule modulated the composition of the intestinal microbiota in AIDS diarrhea patients and thus improved immune function and reduced diarrheal symptoms.

## Introduction

Acquired immunodeficiency syndrome (AIDS) is caused by human immunodeficiency virus (HIV) infection and is characterized by immune system damage and opportunistic infection (Monaco et al., 2016; Nixon et al., 2017). Recent studies have shown that non-AIDS complications are related to the composition and proportion of organisms in the intestinal microbiota, and chronic abnormal immune activation caused by intestinal microbiota translocation (Bandera et al., 2018; Luján et al., 2019). The increase in the incidence and mortality of HIV-related diarrhea is due to changes in intestinal microbes, destruction of the intestinal mucosal barrier, damage to body and lymph tissue, and the loss of CD4^+^ T cells after HIV infection (Lima et al., 1997). Further research also revealed that if CD4^+^ Th17 helper cells are lost early in HIV infection, corresponding changes in the gut microbiota will inevitably occur. Therefore, translocation of the gut microbiota and the inhibition of the regulatory cell (Treg) response are closely related to impaired immune system function (Cunningham-Rundles et al., 2011). As the disease progresses, the gut microbiota in the body also changes. Some studies have shown that when the abundance of *Clostridium* in HIV-infected diarrhea patients decreases, the endocrine interleukin-17 (IL-17) concentration in CD4^+^ T-cell in the body also decreases, leading to disruption of the body’s immune system and deterioration of the body’s inflammatory state; this effect causes a sharp increase in *Escherichia coli* (*E. coli*) abundance in the body and increases the mortality of HIV-infected diarrhea patients (Chui and Owen, 1994; Dillon et al., 2016).

With increased understanding of the relationship between the intestinal microbiota and AIDS, a series of AIDS treatment measures using modern medicine and traditional Chinese medicine targeting the intestinal microbiota have shown efficacy (Wang and Zou, 2011; Liu et al., 2013). Modern medicines regulate AIDS mainly by supplementing with additional intestinal microorganisms and transplanting fecal bacteria to supplement the intestinal microbiota (Cunningham-Rundles et al., 2011; Geng et al., 2020). Supplementing probiotics is beneficial not only for increasing the number of CD4^+^ cells in patients, but also for promoting the recovery of Th17 cells (Blázquez-Bondia et al., 2022). The colonization of the body by probiotics can also help regulate the composition of the host gut microbiota, repair intestinal epithelial barrier damage, reduce intestinal permeability, and enhance intestinal immune function, thereby reducing patient inflammation and improving immune status and prognosis (Blázquez-Bondia et al., 2022). Fecal microbial transplantation (FMT) is a therapeutic method in which the functional community in the feces of healthy people is transferred to AIDS patients through colonoscopy to restore the intestinal microecological balance (Vujkovic-Cvijin et al., 2017). In addition, some researchers have begun to investigate the mechanisms of action of traditional Chinese medicines (TCMs) in the treatment of HIV-infected diarrhea patients (Cohen et al., 2000). TCMs can significantly reduce the amount of fiber and protein fermented in the gastrointestinal tract and utilize the *Lactobacillus* and fiber in the body to produce short-chain fatty acids (SCFAs) and amino acids (glutamine and arginine), thereby effectively improving the immune and metabolic function of HIV-infected patients with diarrhea (Qing et al., 2019). Moreover, traditional Chinese medicine can promote Treg activity, inhibit the production of proinflammatory immune factors, regulate inflammatory enteritis, prolong the survival of HIV-infected diarrhea patients, and thereby reduce the mortality rate of those patients (Chang et al., 2015).

The Xielikang capsule is a TCM used in the AIDS Treatment Project in Henan Province. This medicine was developed by Professor Li Fazhi based on his clinical experience; Xielikang is composed of ingredients such as garlic, nutmeg and gallnut, with garlic oil being the main medicinal component. Xielikang is extracted from garlic and the main component is allicin. Xielikang has the functions of relieving diarrhea with astringents, strengthening the spleen and kidney, and curing diarrhea and dysentery. A previous study revealed that Xielikang has a significant effect when used for the treatment of AIDS-related diarrhea, but the mechanism of its effect on AIDS-related diarrhea has not been determined (Zhang et al., 2017). Therefore, through a randomized, positive drug control test method, patients who passed the inclusion, exclusion and eliminate criteria (Supplementary material S1) were grouped, and 13 healthy volunteers, 18 AIDS patients without diarrhea and 18 AIDS patients with diarrhea were selected. High-throughput sequencing technology was used to investigate variations in the gut microbiota of healthy individuals (ZC, *n* = 13), AIDS patients without diarrhea (FN, *n* = 18), and AIDS patients with diarrhea before and after Xielikang treatment (FA, *n* = 18; FB, *n* = 18). Our objective was to identify the microbiota associated with AIDS-related diarrhea and examine the mechanism of Xielikang treatment of AIDS-related diarrhea.

## Materials and methods

### Case selection and grouping

In this study, we adopted a randomized, positive drug control test method. AIDS patients with diarrhea and spleen and kidney yang deficiency syndrome from the outpatient ward of the AIDS Clinical Research Center of the First Affiliated Hospital of Henan University of Traditional Chinese Medicine (the second ward of infectious diseases) and the AIDS Project of Traditional Chinese Medicine in Henan Province were selected. The study design included 18 AIDS patients with diarrhea, which were divided into the FA group (before treatment) and the FB group (after treatment). In addition, 18 AIDS patients without diarrhea (FN group) and 13 healthy volunteers (ZC group) were used as controls in this study. The clinical data of each group are shown in Table 1.

**Table 1:** Clinical data of each group.

Clinical data	ZC (*n* = 13)	FN (*n* = 18)	FA (*n* = 18)	FB (*n* = 18)	*p* value
Age (year)	48.9 ± 6.3	49.5 ± 11.9	52.0 ± 8.9	52.0 ± 8.9	0.96
Gender (Male/Female)	7/6	15/3	14/4	14/4	0.156
BMI (kg/m^2^)	23.6 ± 2.4	22.8 ± 1.6	23.7 ± 2.3	23.75 ± 2.5	0.145
Diet					
Coarse cereals					0.864
^a^Frequent	2 (15.4%)	3 (16.7%)	1 (5.5%)	1 (5.5%)	
^b^Occasional	9 (69.2%)	11 (61.1%)	14 (77.8%)	14 (77.8%)	
^c^Rare	2 (15.4%)	4 (22.2%)	3 (16.7%)	3 (16.7%)	
Vegetable					0.954
Frequent	4 (30.8%)	5 (27.8%)	3 (16.7%)	3 (16.7%)	
Occasional	8 (61.5%)	11 (61.1%)	13 (72.2%)	13 (72.2%)	
Rare	1 (7.7%)	2 (11.1%)	2 (11.1%)	2 (11.1%)	
Fruit					0.639
Frequent	5 (38.5%)	2 (11.1%)	3 (16.7%)	3 (16.7%)	
Occasional	7 (53.8%)	13 (72.2%)	13 (72.2%)	13 (72.2%)	
Rare	1 (7.7%)	3 (16.7%)	2 (11.1%)	2 (11.1%)	
Oily foods					0.941
Frequent	3 (23.1%)	2 (11.1%)	2 (11.1%)	2 (11.1%)	
Occasional	9 (69.2%)	15 (83.3%)	14 (77.8%)	14 (77.8%)	
Rare	1 (7.7%)	1 (5.6%)	2 (11.1%)	2 (11.1%)	
Smoking	5 (38.5%)	8 (44.4%)	7 (38.9%)	7 (38.9%)	0.981
Drinking	3 (23.1%)	4 (22.2%)	3 (16.7%)	3 (16.7%)	0.945
Transmission route	Not Applicable				Not Applicable
Blood transmission		12 (66.7%)	13 (72.2%)	13 (72.2%)	
Heterosexual behavior		6 (33.3%)	5 (27.8%)	5 (27.8%)	
Homosexual behavior		0 (0.0%)	0 (0.0%)	0 (0.0%)	
Intravenous drug injection		0 (0.0%)	0 (0.0%)	0 (0.0%)	

a. Frequent indicated ≥ 5 times per week. b. Occasional indicated 2–4 times per week. c. Rare indicated ≤ 1 time per week.

### Therapeutic administration methods

The treatment group was given the following medications: Xielikang capsules, produced by Henan Olinte Pharmaceutical Factory (batch number: 20110913; dosage form: capsules; specification: 0.5 g/capsule; usage: 1.5 g/time and 3 times per day, orally). The treatment course lasted 2 weeks. If the patient had a clear intestinal opportunistic infection, the necessary basic treatment was carried out according to the AIDS Common Opportunistic Infections Diagnosis and Treatment Guidelines (Trial) of Henan Province. If the patient had severe dehydration, electrolyte disorders, etc., the corresponding symptomatic treatment was given.

### Sample collection

**Figure 1:**
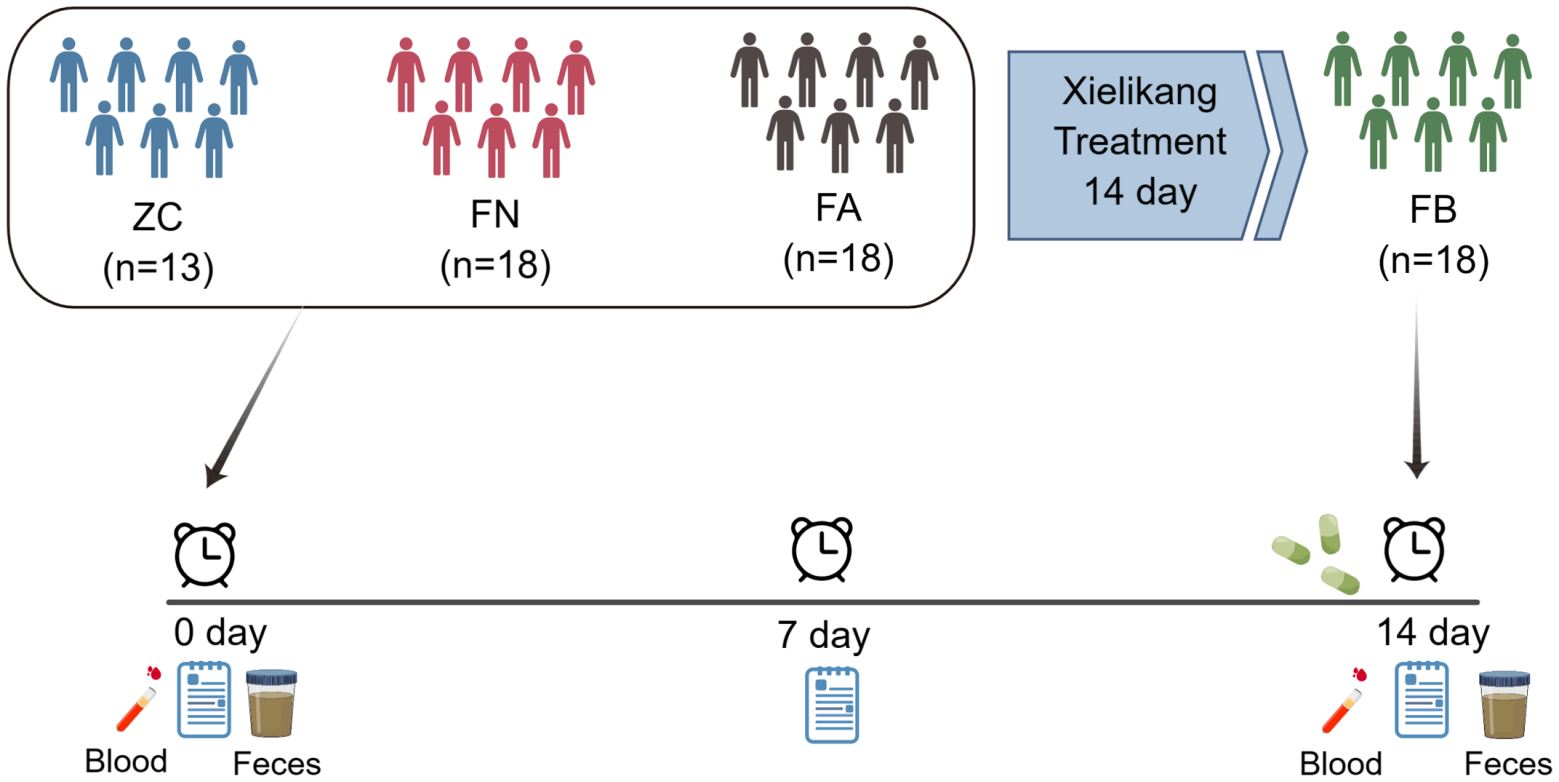
Experimental flowchart.

In the morning, venous blood from the elbow of the subjects was collected and placed in EDTA anticoagulant tubes, and some of the samples were subjected to routine testing (routine blood, liver function and kidney function tests). According to sample collection standards, morning feces were collected from subjects, and approximately 2 g of middle-stage feces was collected and stored in a fecal collection tube with the corresponding amount of fecal preservation solution. The experimental process was shown in Figure 1. The fecal collection tube was placed in liquid nitrogen and stored in a − 80°C freezer in a timely manner for subsequent testing and analysis.

### Cytokine detection

An ELISA kit (Meimian, Jiangsu, China) was used according to the manufacturer’s instructions to determine the levels of inflammatory factors, including IL-1β, IL-6, IL-23, and TNF-α, in the serum.

### Fecal DNA extraction and database construction

A Qiagen fecal DNA extraction kit was used to extract DNA from each fecal sample, and the quality and concentration of DNA were determined via agarose gel electrophoresis and an ultramicro spectrophotometer NanoDrop2000, respectively. Qualified DNA was stored using Illumina’s TruSeq Nano DNA LT Library Kit.

### NovaSeq high-throughput sequencing and bioinformatics analysis

For qualified libraries, a MiSeq Reagent Kit V3 (600 cycles) was used for PE250 sequencing on MiSeq devices. The highly variable V3-V4 region of the bacterial 16S rRNA gene with a length of approximately 468 bp was used for sequencing. The specific primers used were 383F (5′-barcode-ACTCCTACGGGGGGCAGCA-3′) and 806R (5’-GACTACHVGGGTWTCTAAT-3′), which were synthesized by Shanghai Personal Biotechnology Co., Ltd. The sequencing work was carried out by Shanghai Personal Biotechnology Co., Ltd. QIIME2 2019.4 was utilized, and this process was modified and improved according to the official tutorial to analyze microbial bioinformation.^1^ The original sequence data were decoded using the Demux plugin, primed using the Cutadapt plugin, and then processed using the DADA2 plugin for quality filtering, denoising, splicing, and chimerism removal.

(1) The alpha diversity indices were determined,^2^ including the observed species, Shannon, Simpson, Faith’s PD, and Pielou’s evenness indices. (2) Species composition was determined by analyzing the flattened ASV/OTU table, and the specific composition of microbial communities in each sample at each classification level was obtained. The QIIME2 analysis platform was used to draw a bar chart depicting the microbial communities at the phylum level. (3) Cluster analysis was performed using the uclust function of the stat package in R language; the unweighted pair-group method using arithmetic averages (UPGMA) algorithm (i.e., average clustering method) was used by default to perform non-metric multidimensional scaling (NMDS) analysis based on the Bray-Curtis distance matrix, and the differences in microbial community composition were displayed through a two-dimensional sorting graph. (4) The QIIME2 analysis platform was used to perform random forest analysis of the non-flattened ASV/OTU table by calling the “classify_samples_ncv” function in the q2 sample classifier.

### PICRUST2 functional prediction analysis of intestinal microbiota

The phylogenetic investigation of communities by reconstruction of unobserved states (PICRUST2) analysis was performed. First, an evolutionary tree was constructed based on the 16S rRNA gene sequence of the known microbial genome. According to the copy number of the gene family corresponding to the reference sequence in the evolutionary tree, the copy number of the gene family was obtained. By combining the abundance of each sample’s characteristic sequence, the copy number of each sample’s gene family was calculated. Finally, the obtained data was compared and analyzed with databases to determine the abundance of proteins in each metabolic pathway.

### Correlation analysis of different microbiota and cytokines

To analyze the correlation between microbiota and cytokines in the four groups (Pearson correlation), the OmicShare Tools^3^ online analysis software was used; specifically we analyzed the correlation between the top 10 genera and cytokines in the ZC, FN, FA, and FB groups.

### Statistical analysis

This study used a randomized, positive drug controlled trial method, with the results expressed as the mean ± standard error (x ± s). Multiple group comparisons were conducted using one-way ANOVA, with *p* < 0.05 being considered significant and *p* < 0.01 being considered extremely significant. The statistical analysis software used in this study was IBM SPSS 24.0.

## Results

### Diarrhea and cytokines levels

**Figure 2:**
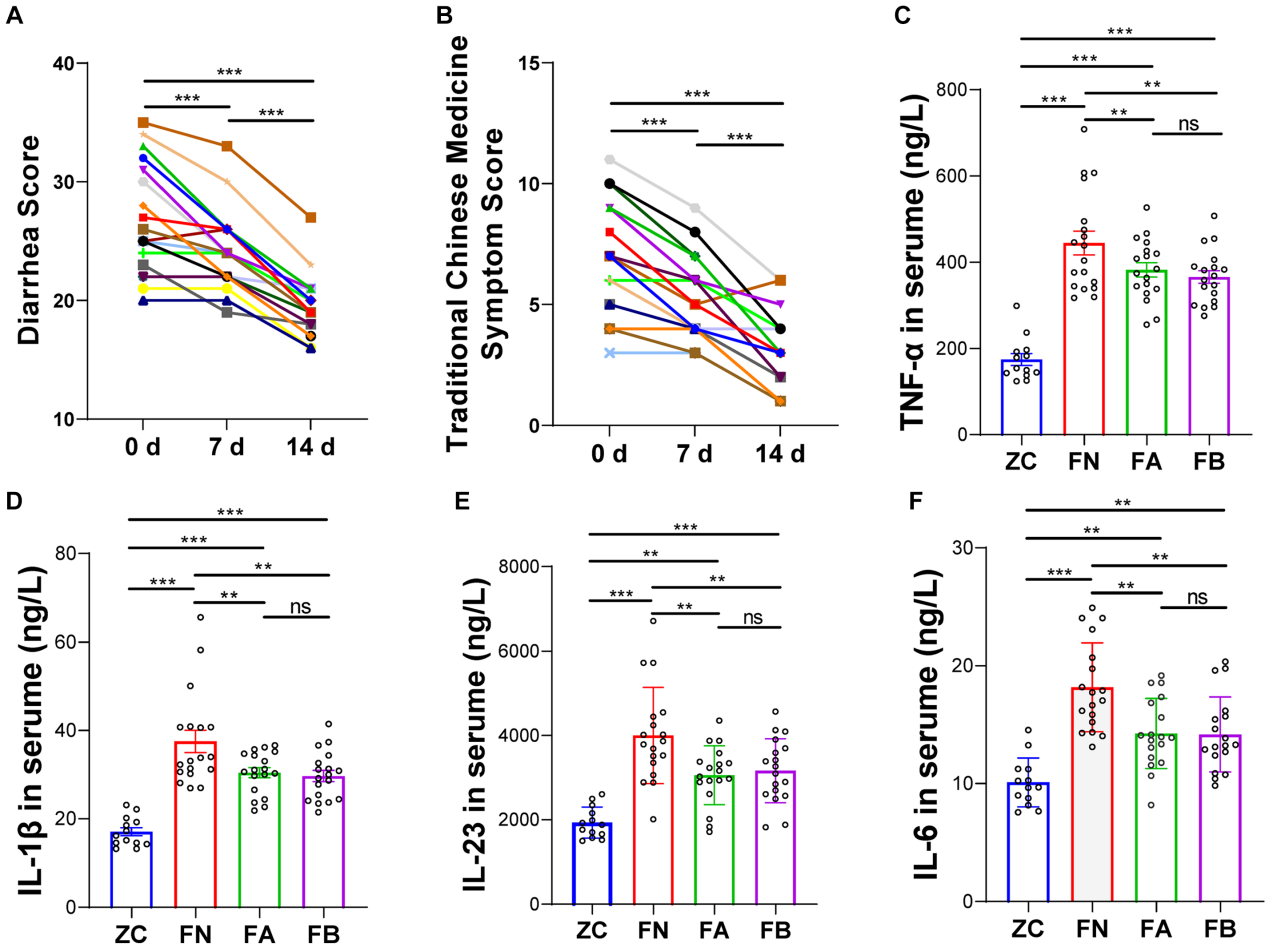
Diarrhea score and the levels of inflammatory factors in AIDS patients with diarrhea. **(A)** Diarrhea score. **(B)** Diarrhea traditional Chinese Medicine (TCM) syndrome score. **(C–F)** Inflammatory factor levels in the serum.

After taking Xielikang for 2 weeks, the diarrhea score and TCM syndrome score of AIDS patients decreased significantly (*p* < 0.01; Figures 2A,B). The level of cytokines in the serum of AIDS patients with diarrhea before and after treatment was significantly higher than that of the healthy control group (*p* < 0.01), but significantly lower than that of AIDS patients without diarrhea (*p* < 0.01). However, there was no significant difference in the levels of inflammatory factors in patient feces before and after treatment with Xielikang (Figures 2C–F).

### Intestinal microbiota diversity

**Figure 3:**
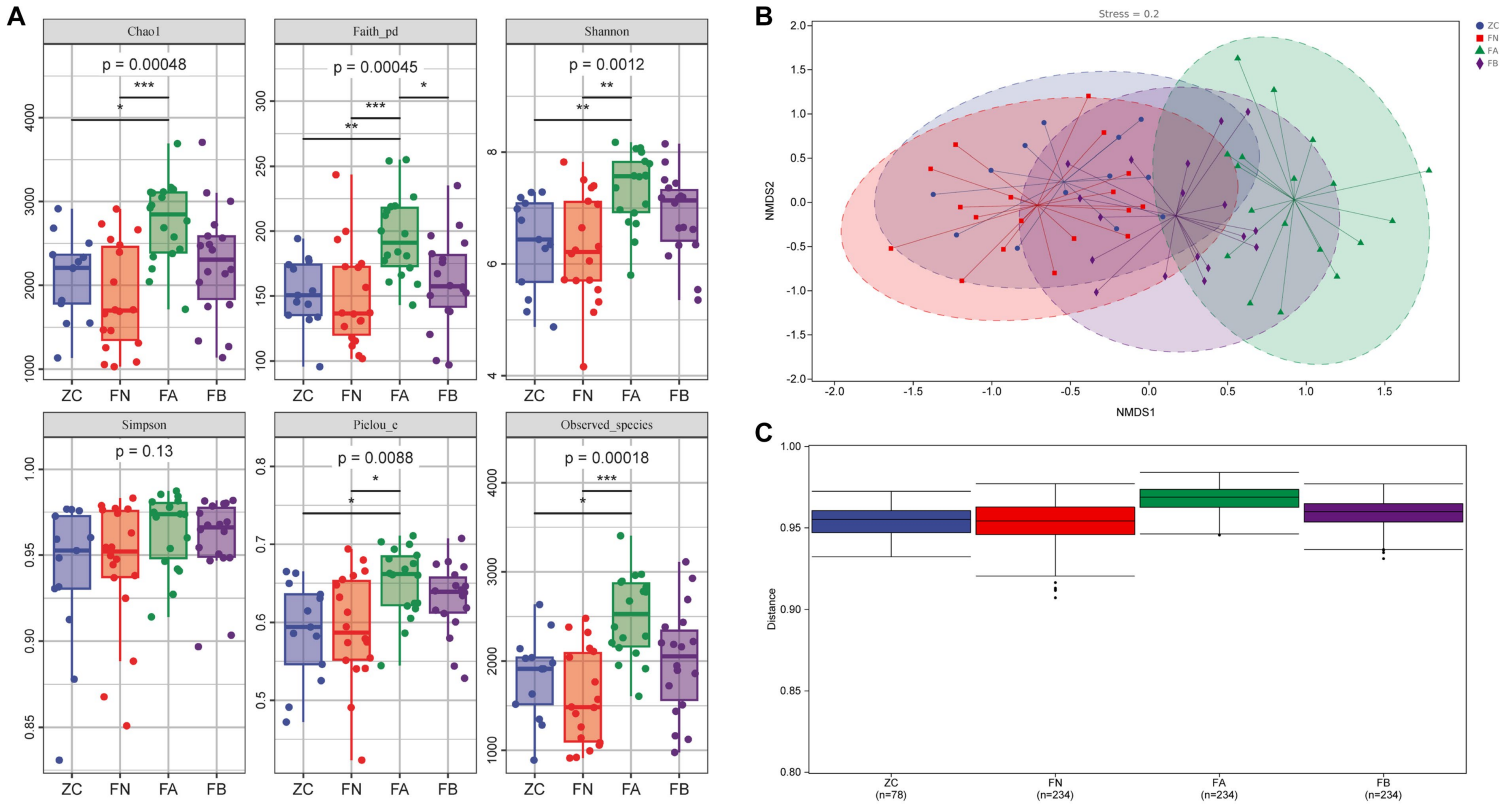
Diversity of the intestinal microbiota. **(A)** Alpha diversity (Chao1, Faith_pd, Shannon, Simpson, Pielou_e and Observed_species indices). **(B,C)** Beta diversity (NMDS analysis and analysis of intergroup differences).

The alpha diversity analysis revealed that the Chao1, Faith_pd, Shannon, Simpson, Pielou_e, and Observed_species indices of the FA group were significantly higher than those of the ZC and FN groups (*p* < 0.05 or *p* < 0.01). The Chao1, Faith_pd, Shannon, Pielou_e, and Observed_species indices of the FB group treated with the Xielikang capsules significantly decreased or exhibited a downward trend (*p* < 0.05 or *p* < 0.01; Figure 3A). The NMDS results revealed significant differences in the composition of the bacterial community among the four groups. The main manifestation of this difference was a small difference in the bacterial composition of the ZC, FN, and FB groups treated with the Xielikang capsules; meanwhile, there was a significant difference in the bacterial composition of the FA group compared to that of the other three groups (Figure 3B).

### Intestinal microbiota composition

**Figure 4:**
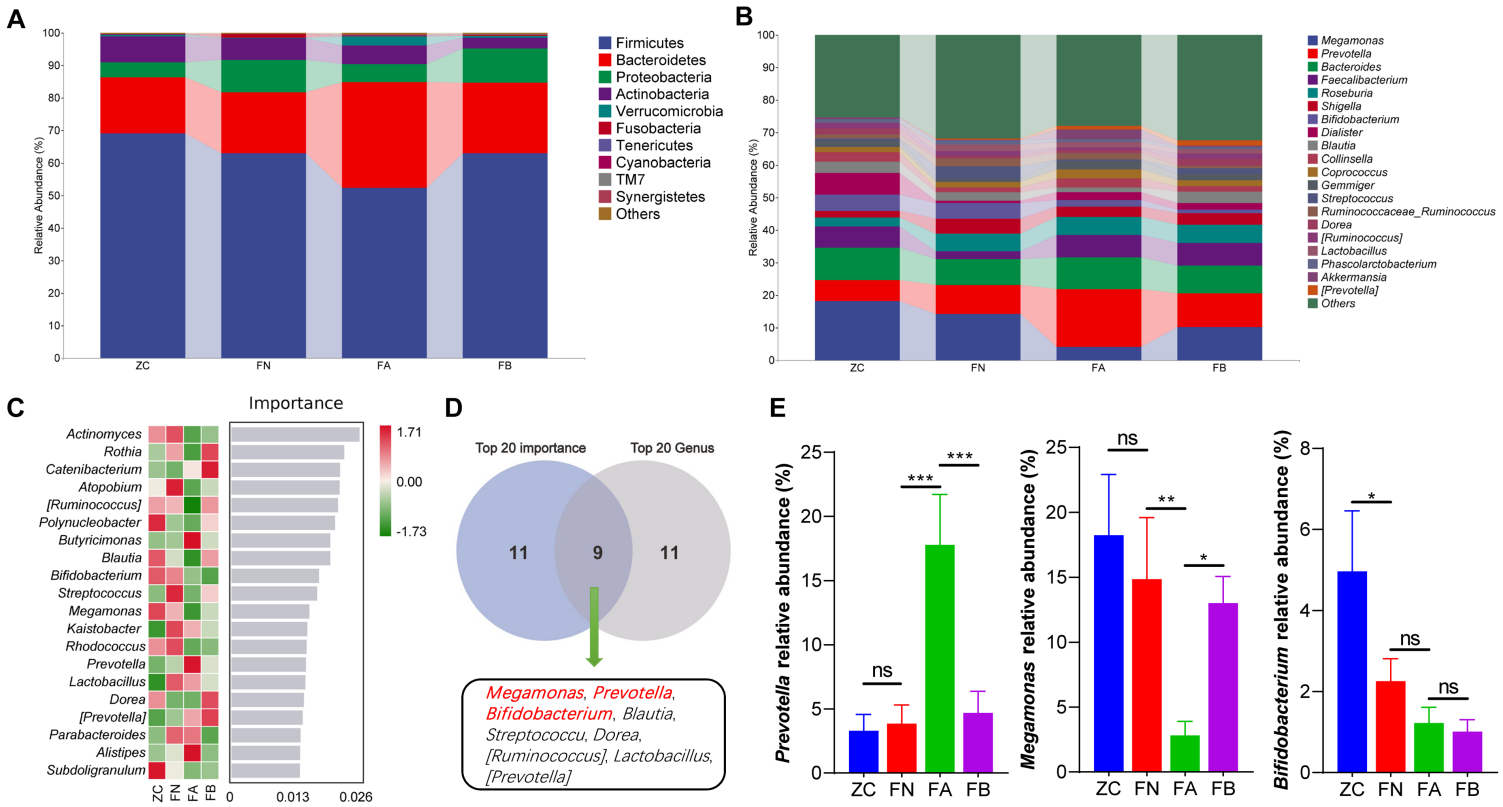
Species composition of the intestinal microbiota. **(A)** The species composition at the phylum level. **(B)** The species composition at the genus level. **(C)** Random forest analysis. **(D)** Interaction analysis of the top 20 important bacterial genera and the top 20 abundant bacterial genera. **(E)** Differences in the abundances of *Megamonas*, *Prevotella* and *Bifidobacterium* in the different groups.

At the phylum level, compared with those in the ZC and FN groups, the relative abundances of Bacteroidetes and Verrucomicrobia in the FA group were significantly increased, while the relative abundance of Firmicutes was significantly decreased compared to that of the ZC and FN groups (*p* < 0.05 or *p* < 0.01). After treatment with Xielikang capsules, the relative abundances of Bacteroidetes and Firmicutes in the FB group were significantly increased (*p* < 0.05 or *p* < 0.01), while the relative abundance of Firmicutes was significantly decreased compared to that in the FA group (*p* < 0.05 or *p* < 0.01); moreover, there was no significant difference when comparing the ZC and FN groups (*p* > 0.05; Figure 4A). At the genus level, compared with those in the ZC and FN groups, the relative abundances of the *Prevotella* and *Shigella* genera in the FA group were significantly increased, while the relative abundances of *Megamonas* and *Bifidobacteria* were significantly decreased compared with those in the ZC and FN groups. After treatment with Xielikang capsules, the relative abundances of *Prevotella* and *Shigella* in the FB group was significantly decreased, while the relative abundances of *Megamonas* and *Bifidobacteria* was significantly increased compared to that in the FA group; moreover, there was no significant difference between the ZC and FN groups (Figures 4B,E). The results of random forest analysis showed that *Actinomyces*, *Rothia* and *Catenibacterium* might be marker species of the differences between groups (Figure 3C). The top 20 most important genera identified by random forest analysis were cross analyzed with the 20 genera with the highest relative abundances, and the nine common genera were *Megamonas*, *Prevotella*, *Bifidobacterium*, *Blautia*, *Streptococcus*, *Dorea*, *[Ruminococcus]*, *Lactobacillus* and *[Prevotella]* (Figures 4C,D).

### Correlations between different intestinal microbiota and cytokine levels

**Figure 5:**
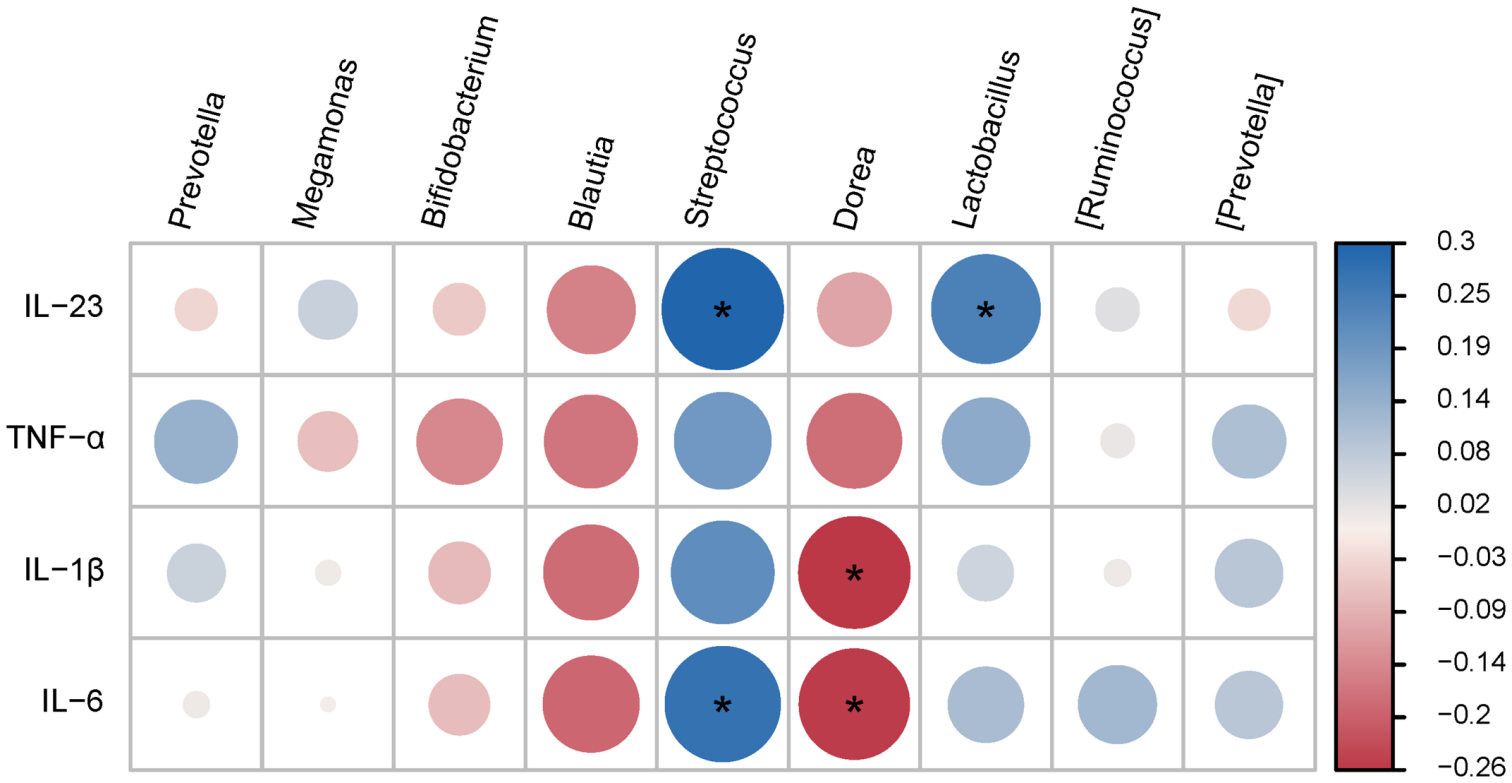
Interactive analysis of the intestinal microbiota and inflammatory factors.

Correlation analysis of the top 10 microbial populations (at the genus level) with inflammatory factors revealed that *Blautia* and *Dorea* were positively correlated with inflammatory factors (IL-1β and IL-6), while *Bifidobacterium*, *Streptococcus* and *Lactobacillus* were negatively correlated with inflammatory factors (IL-23 and IL-6). However, *Prevotella*, *Megamonas*, *[Ruminococcus]* and *[Prevotella]* were not correlated with these inflammatory factors (Figure 5).

### Functional prediction of the intestinal microbiota

**Figure 6:**
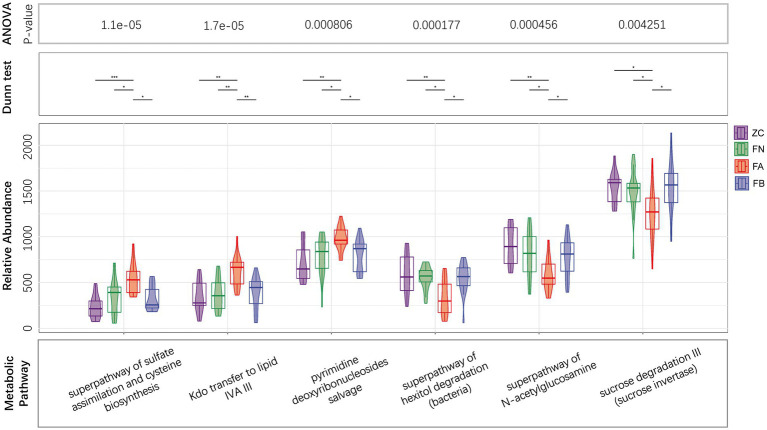
Differences in metabolic pathways mediated by the intestinal microbiota.

**Figure 7:**
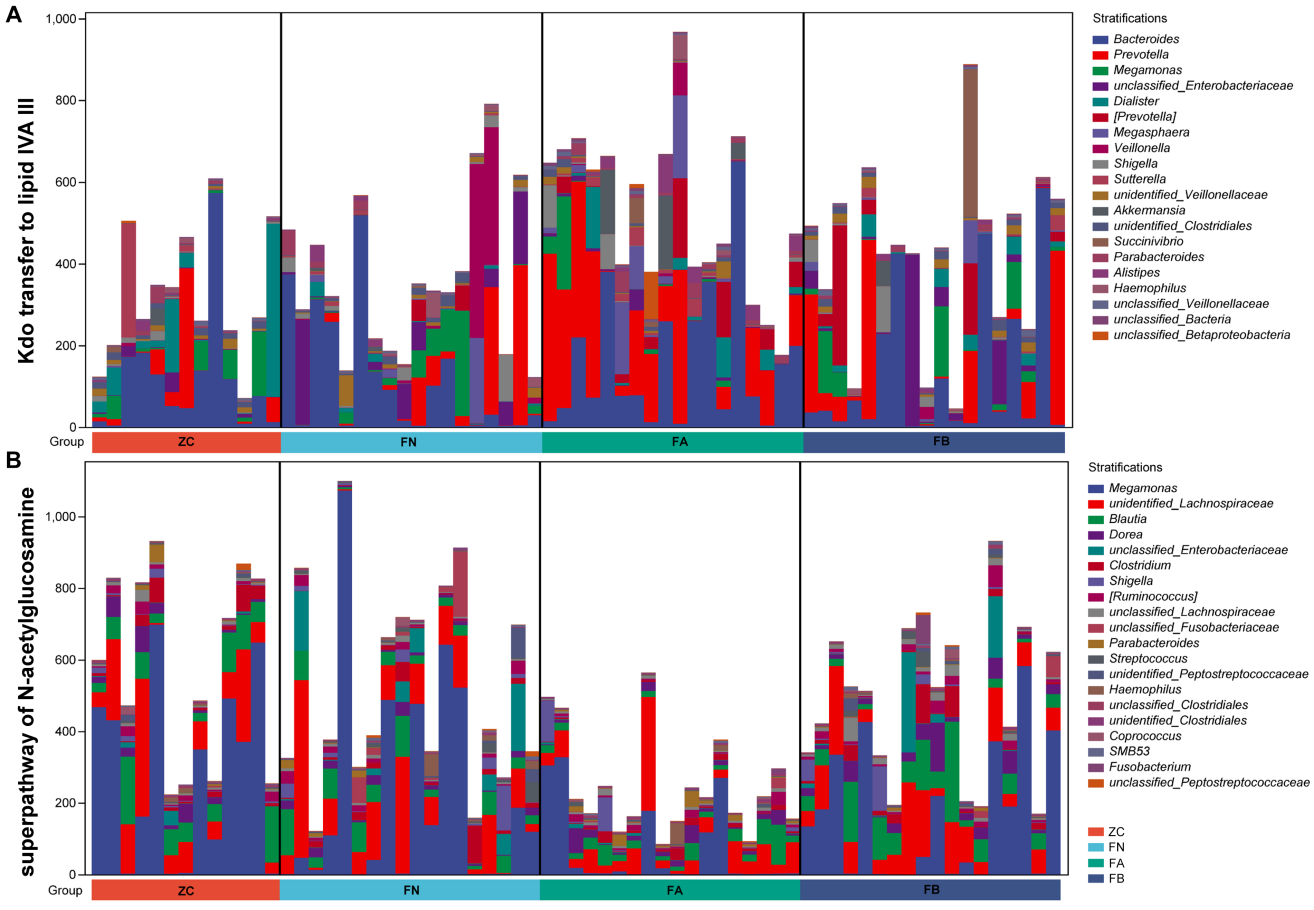
The composition of species involved in **(A)** Kdo transfer to lipid IVA III and **(B)** the superpathway of N-acetylglucosamine pathways.

The results of PICRUST2 functional prediction showed significant differences in the metabolic pathways associated with the four microbial communities. Metabolic pathways associated with the intestinal microbiota of the FA group, such as the superpathway of subduction assessment and cycline biosynthesis, Kdo transfer to lipid IVA III, and pyridine deoxyribonucleoside salvage, were significantly upregulated, while the superpathway of hexitol degradation (bacteria), the superpathway of N-acetylglucosamine, and cross degradation III (cross inversion) were significantly decreased compared to those in the ZC and FN groups. After treatment with Xielikang capsules, the relative abundance of proteins in the metabolic pathways mediated by the FB microbiota was restored (Figure 6). Subsequently, a stratified metabolic pathway abundance table was used to analyze the composition of bacteria that play a role in Kdo transfer to lipid IVA III and the superpathway of N-acetylglucosamine metabolic pathways. In the Kdo transfer to lipid IVA III metabolic pathway, the FA group had a significantly increased relative abundance of *Prevotella*, while the FB group had a decreased relative abundance of *Prevotella* (Figure 7A). In the superpathway of N-acetylglucosamine metabolic pathways, the FA group had a significantly increased relative abundance of *Megamonas*, while the FB group had a decreased relative abundance of *Megamonas* (Figure 7B).

## Discussion

A previous study revealed that 80% of the HIV virus in AIDS patients resides in intestinal tissue, while the viral load in the blood accounts for 2–5% of the total viral load, thus indicating that the intestine is important for HIV infection (Epple and Zeitz, 2012). There are many microorganisms that inhabit the intestines, and under normal circumstances, the gut microbiota is interdependent with the host and external environment and they have a healthy coordinated and balanced relationship (Winston and Theriot, 2020). On the one hand, the host provides conditions for normal colonization of the intestinal microbiota and provides nutrients and an energy (Markowiak and Śliżewska, 2017). On the other hand, the gut microbiota participates in the host’s gastrointestinal digestion, assists in the synthesis and absorption of nutrients, and provides immune regulation, energy supply, information transmission, and defense against diseases (Chen et al., 2019). After HIV enters the human body, it destroys CD4^+^ T lymphocytes in the intestinal lymph tissue, damages the intestinal mucosal barrier, and increases the permeability of the intestinal mucosa, thus disrupting microenvironment stability within the intestinal microbiota and leading to changes in the diversity, quantity, location and structure of the intestinal microbiota (Tincati et al., 2016; Luján et al., 2019; Veazey, 2019; Sainz et al., 2020).

The results of this study showed that the intestinal microbiota of AIDS patients with diarrhea was different from that of AIDS patients without diarrhea and healthy people. Both AIDS and diarrhea symptoms have certain effects on the composition and structure of the intestinal microbiota in humans. The abundance of *Prevotella* and *Bacteroides* in the intestinal microbiota of AIDS patients with diarrhea was higher than that in the intestinal microbiota of AIDS patients without diarrhea and healthy individuals. An increase in the abundance of *Prevotella* increases the pH in the patients’ gut, leading to a more favorable gut environment for HIV infection and replication, and increasing the likelihood of colonization and the amplification of passing bacteria (Meng et al., 2023). Bacteroidetes are the dominant gut bacteria, and the ratio of Bacteroidetes to Firmicutes is closely related to the diversity of the gut microbiota. This ratio is commonly used to measure whether there is an imbalance in the gut microbiota, and an increase in Bacteroidetes abundance suggests that the occurrence of diarrhea in patients is related to an imbalance in the gut microbiota (Elizaldi et al., 2019). In addition, regardless of whether AIDS-positive patients had diarrhea symptoms, compared with those of healthy individuals, the abundance of *Shigella*, *Proteobacteria* and *Enterobacteriaceae* increased, and the content of *Bifidobacteria* and fecal bacteria decreased. *Shigella* species are pathogenic bacteria, and an increase in their abundance is the main driver of diarrhea in AIDS patients. *Proteobacteria* and *Enterobacteriaceae* are conditional pathogens that can also induce inflammatory responses in humans. An increase in the content of these proteins are related to damage to the intestinal mucosa caused by HIV infection, disorder of the intestinal environment, and the persistence of the inflammatory induced immune response (Crowell et al., 2016). *Bifidobacterium* is beneficial to the intestinal tract, while the butyrate produced by fecal bacteria has an anti-inflammatory effect and can regulate human intestinal immunity (Hidalgo-Cantabrana et al., 2017).

A comparative analysis of the intestinal microbiota of AIDS patients revealed that, compared with that of healthy individuals, the intestinal microbiota of AIDS patients had a reduced abundance of *Clostridium* spp. such as *Enterococcus* spp. and *Faecaococcus* spp., and the abundance of bacteria in the microbiota that produce butyrate and participate in tryptophan metabolism in the intestinal tract was reduced (Desai and Landay, 2018; Vujkovic-Cvijin and Somsouk, 2019); furthermore, the proportions of *Prevotella* and *Enterobacteriaceae* were increased (Amador-Lara et al., 2022). The bacteria with high abundance in AIDS patients were mostly pathogenic bacteria (Lewy et al., 2019). Dillon et al. reported that the colonic mucosa of HIV-1 infected individuals was enriched in *Proteobacteria* and *Prevotella,* the abundance of *Helicobacter* and *Klebsiella* significantly increased compared to that in healthy individuals, and the relative abundance of Firmicutes and Bacteroides decreased (Dillon et al., 2014). Pathogenic bacteria and endotoxins attack the intestinal mucosa, thereby increasing intestinal permeability, damaging the membrane barrier, stimulating the release of histamine, 5-HT, prostaglandin, tryptase and other active substances, and accelerating intestinal peristalsis (D'Angelo et al., 2017; Villanueva-Millán et al., 2019; Nganou-Makamdop et al., 2021).

The Xielikang capsule has astringent bowel and antidiarrheal effects, and invigorates the spleen and kidney, kills pathogens and prevents dysentery (Zhang et al., 2017). Previous studies have confirmed that this medicine has good clinical efficacy in AIDS patients with diarrhea, and treatment significantly reduces the clinical symptoms of patients, increases the level of intestinal SIgA, reduces the imbalance in the intestinal microbiota, and improves the quality of life of patients (Zhang et al., 2017). Diarrhea, which is a common complication of AIDS, is mostly caused by damage to the gastrointestinal mucosa and immune function, and diarrhea can also lead to weight loss and malnutrition. This condition has a serious impact on the quality of life of AIDS patients and is also one of the main causes of death (Castro and Chin-Beckford, 2015).

When the effect of the Xielikang capsule on the intestinal microbiota of patients with AIDS and diarrhea was evaluated, the results showed that there were no significant differences in the diversity, richness, evenness or composition of the intestinal microbiota before or after treatment with Xielikang (*p* > 0.05), but there were differences in the species composition. After treatment with Xielikang, the intestinal microbiota of patients was enriched in Firmicutes and Bacteroides. The relative abundances of Faecalibacterium and Roseburia increased. Normal proportions of Firmicutes and Bacteroides protect the intestinal mucosa and help maintain the intestinal microbiota balance and normal physiological functions to prevent and treat diarrhea (Grigor'eva, 2020).

The mucus is an important component of the intestine that helps to separate luminal bacteria from the host epithelium and form a barrier (Luis and Hansson, 2023). Symbiotic bacteria degrade and utilize mucins and O-glycans by encoding multiple proteins, which in turn affects the intestinal colonization of symbiotic bacteria. Normal interactions between symbiotic bacteria and the mucus layer are crucial for maintaining health (Birchenough et al., 2023). In this study, we found that the relative abundance of *Prevotella* in the intestine of AIDS patients with diarrhea was significantly higher than that in the intestine of AIDS patients without diarrhea. *Prevotella* has glycosylhydrolases that can degrade mucin (Glover et al., 2022). An excessive abundance of *Prevotella* exacerbates the consumption of mucus, which gives some pathogenic microorganisms such as *Shigella* the opportunity to directly contact intestinal epithelial cells, thereby damaging intestinal epithelial cells and leading to gastrointestinal symptoms such as diarrhea. However, in this study, we used Xielikang to treat AIDS patients with diarrhea and found that Xielikang capsule can significantly alleviate the clinical symptoms (diarrhea) of AIDS patients with diarrhea and adjust the intestinal microbiota (by restoring the relative abundance of *Prevotella* and *Megamonas* to the level of AIDS patients without diarrhea and even that of healthy people). These findings indicate that the function of Xielikang capsules may be to regulate the intestinal microbiota imbalance in AIDS patients (by reducing the consumption of mucus by *Prevotella* and restoring the barrier function of mucus), reestablish the intestinal microecology balance, and improve the immune function of the body, thereby enhancing the first line defense of the intestinal mucosa and improving and protecting intestinal function. In the long run, exercise can improve patients’ physical fitness, restore physical strength, and prolong their survival time. In addition, the results of PIGRUST2 functional prediction further indicated that treatment with Xielikang capsules significantly reduced Kdo transfer to lipid IVA III and increased the relative abundance of metabolic pathways, such as the superpathway of N-acetylglucosamine (to the level of the AIDS patients without diarrhea or even that of healthy group). Therefore, the efficacy of Xielikang in treating AIDS patients with diarrhea and improving the intestinal microbiota community structure are achieved by creating a microbial community similar to that of a normal gut, which balances the intestinal microecology and improves immunity.

## Conclusion

In conclusion, the composition and function of the intestinal microbiota change significantly in AIDS diarrhea patients change significantly, which affects the immune function of the host. The Chinese medicine Xielikang can regulate the composition of the intestinal microbiota in AIDS patients with diarrhea and thus improve immune function and reduce diarrheal symptoms.

## Data availability statement

The datasets presented in this study can be found in online repositories. The names of the repository/repositories and accession number(s) can be found at: https://www.ncbi.nlm.nih.gov/genbank/, PRJNA977211.

## Ethics statement

The studies involving humans were approved by the clinical research plan meets the ethical standards and has been approved by the clinical ethics committee of the scientific research project of The First Affiliated Hospital of Henan University of Chinese Medicine: 2018HL-044-01 and 2019HL-099-01. The studies were conducted in accordance with the local legislation and institutional requirements. The participants provided their written informed consent to participate in this study.

## Author contributions

PM: Writing – original draft. GZ: Methodology, Writing – review & editing. XM: Data curation, Writing – review & editing. XD: Data curation, Writing – review & editing. XS: Investigation, Writing – review & editing. SD: Investigation, Writing – review & editing. RY: Investigation, Writing – review & editing. LX: Writing – review & editing, Funding acquisition, Resources, Supervision.

## Footnotes

^1^http://docs.qiime2.org/2019.4/tutorials/

^2^http://scikitbio.org/docs/latest/generated/skbio.diversity.alpha.html#moduleSkbio.diversity.alpha

^3^https://www.OmicShare.com/Tools
