# Supplementary material for: Traditional Chinese medicine (Xielikang) reduces diarrhea symptoms in acquired immune deficiency syndrome (AIDS) patients by regulating the intestinal microbiota
Source: Front Microbiol. 2024 Feb 16;15:1346955. doi: 10.3389/fmicb.2024.1346955 (PMC10904582; doi:10.3389/fmicb.2024.1346955)
Supplement: Supplementary file 2 [file Data_Sheet_2.docx]

1 Research subjects

1.1 Diagnostic criteria

1.1.1 Diagnostic criteria for traditional Chinese medicine

Diagnostic criteria for syndromes associated with deficiency of the spleen and kidney yang in traditional Chinese medicine were formulated with reference to the Guiding Principles for Clinical Research of New Traditional Chinese Medicine ^[1]^ and the Clinical Technical Scheme of Traditional Chinese Medicine for AIDS ^[2]^.

The main symptoms were loose and loose stools, sometimes as if they were watery, diarrhea in the morning, or stubbornness.

Secondary symptoms included cold pain in the navel and abdomen, fatigue and weakness, mental fatigue and lazy speech, cold body and cold limbs, sore and soft waist and knees, light and plump tongue, thin and white fur, and thin and heavy veins.

If there were 2 main symptoms and 2 secondary symptoms, the pulse condition is basically consistent and can be diagnosed.

1.1.2 Diagnostic criteria for Western medicine

① AIDS diagnostic criteria: AIDS-related diarrhea is a general term for a group of diseases in which diarrhea is the main symptom during AIDS (including the acute HIV infection period, asymptomatic period and AIDS period). Therefore, the diagnostic criteria for AIDS were established according to the Guidelines for AIDS Diagnosis and Treatment issued by the Ministry of Health of the Ministry of Health of the People's Republic of China in 2011.

Diagnostic principles: The diagnosis of HIV/AIDS needs to be combined with epidemiological history (including unsafe sex life history, intravenous drug history, blood or blood products imported without receiving an anti-diagnosis of HIV/AIDS, children born to anti-HIV-positive persons or with occupational exposure history), clinical manifestations and laboratory tests to conduct a comprehensive analysis and make a diagnosis carefully. The diagnosis of HIV/AIDS must be confirmed by confirmatory tests, and the detection of HIV RNA and P24 antigens can aid in the diagnosis of HIV/AIDS, especially by shortening the antibody "window period" and helping to diagnose HIV infection in newborns in the early stage.

Acute phase: Patients with a recent epidemiological history and clinical manifestations can be diagnosed by combining laboratory HIV antibodies from negative to positive or by simply testing for HIV antibodies from negative to positive in the laboratory.

In the symptomatic stage, according to the epidemiological history, patients can be diagnosed by combination with anti-HIV-positivity, or patients can be diagnosed only by laboratory tests of anti-HIV-positivity.

AIDS duration: Patients can be diagnosed with AIDS if they have an epidemiological history and a laboratory test confirming that they are positive for HIV antibody plus any of the following items. If the HIV antibody test is positive, AIDS can be diagnosed even if the number of CD4+ T lymphocytes is less than 200/mm^3^.

(1) Persistent irregular fever of unknown cause above 38 ℃ for more than 1 month;

(2) Patients with chronic diarrhea occurring more than 3 times per day for >1 month;

(3) Patients with a weight loss greater than 10% within 6 months;

(4) Recurrent oral candidiasis;

(5) Patients with recurrent herpes simplex virus infection or Shingles virus infection;

(6) Pneumocystis pneumonia (PCP);

(7) Patients with recurrent bacterial pneumonia;

(8) Patients with active tuberculosis or nontuberculous mycobacterial disease;

(9) Patients with deep fungal infection;

(10) Central nervous system-occupying lesions;

(11) Patients with dementia among middle-aged and young people;

(12) Patients with active cytomegalovirus infection;

(13) *Toxoplasma gondii* encephalopathy;

(14) Penicillium infection;

**Inclusion criteria:**

1. The patient was positive for HIV and confirmed to meet the diagnostic criteria for AIDS virus infection by diagnostic tests.

2. CD4+T cells > 200/μL,

3. No symptoms of chronic infections such as fatigue, hot flashes, or night sweats within at least one month; no significant changes in weight; no metabolic or connective tissue diseases; and no use of antibiotics, probiotics, or other preparations in the past month.

4. Expected survival time > 2 years;

5. Patients who voluntarily participated in this study and signed an informed consent form;

6. There was no other sexually transmitted disease, such as syphilis or genital warts.

**Exclusion criteria:**

1. Those who met the diagnostic criteria for HIV infection according to unconfirmed experiments;

2. Those who did not understand the purpose of this study and were unwilling to sign an informed consent form;

3. Those who had taken antibiotics within the past month;

4. Multiple organ damage;

**Elimination criteria:**

1. The selection of cases violates the inclusion criteria;

2. Those who have not been treated according to regulations or whose information is incomplete after inclusion, which affects the evaluation of efficacy or safety judgment;

3. During the research process, the subjects may have taken other therapeutic drugs or used other treatment methods that may have affected the efficacy assessment.

**References**

[1] Zheng Xiaoyu Guiding Principles for Clinical Research of New Traditional Chinese Medicine [M] China Medical Science and Technology Press, 2002

[2] Wei Jian'an, Sun Limin, Wang Jian Clinical technical scheme of traditional Chinese medicine for AIDS treatment (trial) [C] The first national academic conference on infectious diseases of integrated traditional and western medicine, 2006

**Diarrhea score**

|  | **Clinical feature** | **Scoring** |
| --- | --- | --- |
| a. | Forming (not soft in texture, in its original shape) | 0 points |
| b. | Half formed (with a soft texture but still maintaining its original shape) | 2 points |
| c. | Loose (no original shape of stool) | 4 points |
| d. | Water sample (porridge or water shape) | 6 points |

**TCM syndrome**

| **Symptom** | **Clinical feature** | **Grade** | **Scoring** |
| --- | --- | --- | --- |
| **Diarrhea** | None | Normal | 0 points |
|  | Unformed stool, 3-4 times a day | Mild | 2 points |
|  | Loose stools, 5-10 times daily | Moderate | 4 points |
|  | Stool like water, more than 10 times a day | Severe | 6 points |
| **Fear of cold and limb coldness** | None | Normal | 0 points |
|  | Slight coldness in hands and feet | Mild | 2 points |
|  | Significant coldness in limbs | Moderate | 4 points |
|  | Cold all over the body | Severe | 6 points |
| **Burnout and feeble** | None | Normal | 0 points |
|  | Slightly tired body, can persist in light physical work | Mild | 1 points |
|  | Limbs weak, barely adhering to daily activities | Moderate | 2 points |
|  | Full body weakness, unwilling to move around all day long | Severe | 3 points |
| **Anorexia** | None | Normal | 0 points |
|  | Poor appetite, reduced appetite by less than 1/3 | Mild | 1 points |
|  | Poor appetite, reduced appetite by 1/3-1/2 | Moderate | 2 points |
|  | No appetite all day long, reducing appetite by more than half | Severe | 3 points |
| **aching lumbus and knees** | None | Normal | 0 points |
|  | Occasional attacks | Mild | 1 points |
|  | Recurrent attacks | Moderate | 2 points |
|  | Persistent attacks, difficult to alleviate | Severe | 3 points |
| **Epigastric distension** | None | Normal | 0 points |
|  | Postprandial fullness in the epigastric region, self-relieving within 30 points | Mild | 1 points |
|  | Postprandial fullness in the epigastric region, self-relieving within 2 hours | Moderate | 2 points |
|  | Full of abdominal distension all day long | Severe | 3 points |
| **Abdominal distension and pain** | None | Normal | 0 points |
|  | Occasional mild abdominal distension and pain | Mild | 1 points |
|  | Abdominal distension and pain are severe but tolerable | Moderate | 2 points |
|  | Severe abdominal distension and pain cannot be tolerated, medication is needed to control it | Severe | 3 points |
